# Assessment of liver stiffness measurement and ultrasound findings change during inotuzumab ozogamicin cycles for relapsed or refractory acute lymphoblastic leukemia

**DOI:** 10.1002/cam4.4390

**Published:** 2021-12-30

**Authors:** Federico Ravaioli, Giovanni Marconi, Giovanni Martinelli, Elton Dajti, Chiara Sartor, Maria Chiara Abbenante, Luigina Vanessa Alemanni, Jacopo Nanni, Benedetta Rossini, Sarah Parisi, Luigi Colecchia, Gianluca Cristiano, Giovanni Marasco, Amanda Vestito, Stefania Paolini, Francesca Bonifazi, Antonio Curti, Davide Festi, Michele Cavo, Antonio Colecchia, Cristina Papayannidis

**Affiliations:** ^1^ IRCCS Azienda Ospedaliero‐Universitaria di Bologna Dipartimento di Scienze Mediche e Chirurgiche (DIMEC) Università di Bologna Bologna Italy; ^2^ IRCCS Azienda Ospedaliero‐Universitaria di Bologna Istituto di Ematologia “Seràgnoli” Dipartimento di Medicina Specialistica Diagnostica e Sperimentale Università di Bologna Bologna Italy; ^3^ IRCCS Istituto Romagnolo per lo Studio dei Tumori (IRST) “Dino Amadori” Meldola Italy; ^4^ IRCCS Azienda Ospedaliero‐Universitaria di Bologna Istituto di Ematologia “Seràgnoli” Bologna Italy; ^5^ Programma Dipartimentale di Terapie Cellulari Avanzate IRCCS Azienda Ospedaliero‐Universitaria di Bologna Bologna Italy; ^6^ Gastroenterology Unit Department of Medical Specialties, University of Modena & Reggio Emilia and Azienda Ospedaliero‐Universitaria di Modena Modena Italy

**Keywords:** clinical cancer research, clinical management, elastography, leukemia, liver stiffness, risk assessment, ultrasound

## Abstract

In adult patients, acute lymphoblastic leukemia (ALL) is a rare hematological cancer with a cure rate below 50% and frequent relapses. With traditional therapies, patients with relapsed or refractory (R/R) ALL have a survival that may be measured in months; in these patients, inotuzumab ozogamicin (IO) is an effective therapy. IO was linked to increased risk of veno‐occlusive disease/sinusoid obstruction syndrome (VOD/SOS), liver injury, and various grade of liver‐related complications during clinical trials and real‐life settings; however, hepatologic monitoring protocol is not established in this population. In our institution, 21 patients who received IO (median of 6 doses of IO administered) for R/R ALL were prospectively followed for hepatologic surveillance, including clinical evaluation, ultrasonography, and liver stiffness measurement (LSM) biochemistry. After a median follow‐up of 17.2 months, two SOS events were reported (both after allogeneic transplant) as IO potentially related clinically relevant adverse event. Mild alterations were reported in almost the totality of patients and moderate‐severe liver biochemical alterations in a quarter of patients. Within biochemicals value, AST and ALP showed an augment related to IO administration. LSM linearly augmented for each IO course administered. Baseline LSM was related to liver‐related changes, especially with the severity of portal hypertension (PH)‐related complications. Pre‐transplant LSM was higher in patients receiving IO when compared with a control cohort. PH‐related complications were discovered in nearly 77% of patients, with clinically significant PH occurrence and development of ascites in 38% and 14%, respectively. This prospective experience constitutes the rationale to design a hepatologic monitoring program in patients receiving IO. LSM may be of pivotal importance in this program, constituting a rapid and effective screening that quantitatively correlates with liver alterations.

## Graphical abstract

Intensive longitudinal hepatologic monitoring detected subclinical signs of liver injury in leukemia patients treated with inotuzumab. Ultrasound and liver stiffness measurement can help diagnosing/monitoring portal hypertension‐related complications after inotuzumab therapy.

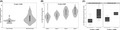

**Lay summary**

Our work changes clinical practice in acute lymphoblastic leukemia patients treated with inotuzumab ozogamicin. We have established proper intensive hepatology monitoring in our patient set. We presented data on several widely available laboratory and imaging methods for assessing liver status. To determinate a precise hepatological assessment will improve the safety and long‐term outcome of this population. Safety concerns related to inotuzumab can be overwhelmed by careful patient evaluation using noninvasive methods.

## 1 INTRODUCTION

In relapsed or refractory B‐cell precursor acute lymphoblastic leukemia (ALL), inotuzumab ozogamicin (IO), a humanized monoclonal antibody‐drug conjugate (ADC) targeting CD22, showed extraordinary efficacy.^1^ Since the first clinical trial using ozogamicin‐based ADCs, an increased risk of mild to moderate liver‐related adverse effects during and after the dosing period has been observed.^1^, ^2^ The development of sinusoidal obstruction syndrome/veno‐occlusive disease (SOS/VOD; formerly known as a veno‐occlusive disease [VOD] of the liver) and liver toxicity was associated with gemtuzumab ozogamicin and IO administration.^3^, ^4^, ^5^ IO was associated with the risk of SOS/VOD, especially in patients who received hematopoietic stem cell transplantation (HSCT) after IO therapy (cumulative incidence of 22%).^6^, ^7^ According to its pathogenic mechanisms, SOS/VOD is portal hypertension (PH)‐related syndrome, also reported in patients who undergo HSCT conditioned with single or de alkilant or who receive methotrexate^8^ and platinum‐based drugs.^9^, ^10^, ^11^, ^12^ In the HSCT setting, the SOS/VOD clinical diagnosis is now established according to the European Society for Blood and Marrow Transplantation (EBMT) clinical criteria, considering the presence of hyperbilirubinemia, painful hepatomegaly, weight gain, and ascites.^13^, ^14^ Hepatic vein pressure gradient (HVPG) measurement and concomitant transjugular liver biopsy (LB) represent an accurate yet invasive approach to correctly assess PH grade and diagnose doubtful SOS/VOD cases.^15^, ^16^

The use of noninvasive liver tests (NITs) such as the liver (LSM) and spleen (SSM) stiffness measurements by transient elastography (TE) has recently been proposed to assess the PH degree and the PH‐related alterations both in the hepatological context^17^ and also in the evaluation of SOS/VOD and other liver and nonliver complications after HSCT.^18^, ^19^, ^20^, ^21^, ^22^, ^23^ Stiffness is not only sensitive to liver fibrosis.^24^ Still, it is also affected by various factors such as congestion, portal hypertension, and inflammation, making its measurement suitable for studying vascular liver diseases and drug‐induced liver injury (DILI).^17^

In the same setting, the role of abdominal grayscale and Doppler ultrasound (US) has been extensively demonstrated as a helpful bedside method to contribute to the differential diagnosis of abdominal complications (cholecystitis, cholangitis, pancreatitis, etc.) and PH‐related complications.^25^, ^26^, ^27^

Thus, we aimed to investigate the role of specialistic hepatic monitoring, including biochemical, LSM, and US findings, in a cohort of patients with ALL to evaluate early hepatobiliary and PH‐related complications during and after the IO therapy.

## 2 MATERIALS AND METHODS

### 2.1 Patients and study design

Here we report a monocentric, prospective, longitudinal study on liver evaluation of patients receiving the label IO at the Istituto di Ematologia "Seràgnoli," Dipartimento di Medicina Specialistica, Diagnostica e Sperimentale, Università degli Studi, Bologna, Italy, from February 2017 to December 2020.

All patients with refractory or relapsed ALL, according to the 2016 World Health Organization criteria^28^ aged 18 years or older, who were indicated to be treated with IO, were prospectively followed for hepatologic surveillance specialistic consultant and included in the analysis.

Patients with any contraindication for IO treatment or treated in a clinical trial or off‐label use were excluded from this study. Furthermore, to avoid possible technical LSM bias, patients with BMI over 40 kg/m^2^, the presence of a pacemaker or implantable device, ascites, or advanced chronic liver disease (ACLD) at screening assessment were also excluded. IO was administered at a dose of 0.8–0.5–0.5 mg/sqm in weekly administration for the first course of therapy, and 0.5–0.5–0.5 mg/sqm in weekly administration for the subsequent courses for up to two courses in patients’ candidate to allogeneic hematopoietic stem cell transplant (one patient received three courses of IO before transplant for a delay in donor matching), for up to six courses in transplant non‐eligible patients. IO was administered as single‐agent therapy and was not combined with other chemotherapy. All patients received ursodeoxycholic acid (300 mg every 12 h) during IO treatment as prophylaxis. All patients gave written informed consent to the label use of inotuzumab and clinical data according to the Declaration of Helsinki, the good clinical practice (GCP), and institutional guidelines. Data were collected in observational studies 012/2009/U/Tess, 01/2011/U/Tess, 10/2011/U/Tess, and 253/2013/O/Tess, in accordance with GCP and as approved by the local ethical review board.

The main aim of the study was to investigate the role of hepatic monitoring by LSM during IO therapy evaluated with TE through a dense evaluation program; the secondary objectives were: (i) to evaluate the role of liver biochemical evaluation in monitoring hepatic alterations in patients undergoing IO therapy; (ii) to estimate the prevalence of VOD/SOD and hepatotoxic and related to portal hypertension events by the US in patients undergoing IO therapy; and (iii) to explore the putative role of LSM assessment in patients treated with IO as bridging therapy prior to HSCT.

The study protocol foresaw a clinical evaluation, biochemical assays, a grayscale, color Doppler and US, and LSM evaluation of all patients included at enrollment (within 21 days prior to the first IO cycle). Then the LSM subsequent evaluations were performed by means of a dense schedule of LSM assessment and biochemical laboratory tests at the end of each IO cycle. At the end of the last IO cycle, an abdominal US was performed. In doubtful cases to confirm the suspect of portal hypertension‐related events and the above schedule of the study protocol, extra liver evaluation, such as liver biopsy, hepatic venous pressure gradient (HVPG), contrast enhancement RM, and upper endoscopy were carried out.

To compare INO‐ and HSCT‐treated patients with an HSCT population not previously treated with IO, we used the recently published cohort of HSCT patients of adult monocentric ELASTOVOD study.^18^

### 2.2 Liver and spleen stiffness measurements

Liver stiffness measurement (LSM) values were assessed by expert operators using the FibroScan^®^ apparatus with the “M” probe (Echosens, Paris, France) after overnight fasting. LSM values were obtained according to the last European Federation of Societies for Ultrasound in Medicine and Biology (EFSUMB) Guidelines and Recommendations on the Clinical Use of Ultrasound Elastography in hepatic and non‐hepatic applications^25^, ^29^; At least 10 measurements with an IQR/M ≤30% were used as reliability criteria. SSM was carried in association with the LSM assessment, with the same probe used to perform LSM using the FibroScan^®^ apparatus, as previously described.^30^ Cut‐offs of 21 kPa and 52.8 kPa were used to define the presence of clinically significant PH (CSPH) by LSM and SSM, respectively.^31^

### 2.3 Abdominal ultrasound

All patients underwent baseline grayscale and color Doppler abdominal ultrasound (US) examinations before the first IO cycle. According to EFSUMB recommendations for the performance and reporting of ultrasound examinations in portal hypertension,^32^ the portal hypertension‐related findings were evaluated. US examination was performed with a Sequoia™, Siemens Acuson with convex probe SC1.

### 2.4 Hepato‐biliary and portal hypertension‐related alterations

According to the monitoring adverse events of the INO‐VATE study,^33^ treatment‐emergent hepato‐biliary alterations comprised adverse events related to liver toxicity and liver‐related investigations that occurred on or after cycle 1, Day 1 but within the last follow‐up visit or the death of the patient. Among these events, SOS/VOD during study treatment or follow‐up without HSCT intervention (60 days after the last dose of IO) and after HSCT treatment was considered. The National Cancer Institute Common Terminology Criteria for Adverse Events (CTCAE version 4.0) showed that hepatobiliary alterations were stratified.^34^ Moreover, a noninvasive diagnostic approach (mainly grayscale and color Doppler abdominal ultrasound and liver and spleen stiffness measurement) was evaluated for portal hypertension‐related signs and complications during and after IO cycles.^35^ When indicated, invasive PH diagnostic approaches (HVPG and upper endoscopy) were performed to confirm/exclude the presence of CSPH.

### 2.5 Data management and statistical analysis

Data were collected and managed using REDCap electronic data capture tools hosted at the University of Bologna, Italy.^36^ Categorical data were expressed as numbers (percentages) and continuous variables as the median and interquartile range (IQR). Due to the small sample size, nonparametric tests were used and preferred.^37^ For group comparison, the Wilcoxon signed‐rank test was used for continuous variables and the Fisher's exact test for categorical variables. Boxplots and violin plots were constructed to present changes in parameters considered during the IO treatment phases. The Spearman rank correlation coefficient (Spearman rho) and its associated probability (*p*) were used to examine the correlation between LS values and hepato‐biliary and portal hypertension‐related alterations. Given the study's exploratory nature and the related limited use of IO, a calculation of the formal sample was not envisaged. Still, a time limit of a maximum of 3 years of enrollment was defined. All *p*‐values refer to two‐tailed tests of significance. Statistical analyses were performed using Stata/SE (version 16; Stata Corp) for Windows.

## 3 RESULTS

### 3.1 Patients and treatments

**FIGURE 1:**
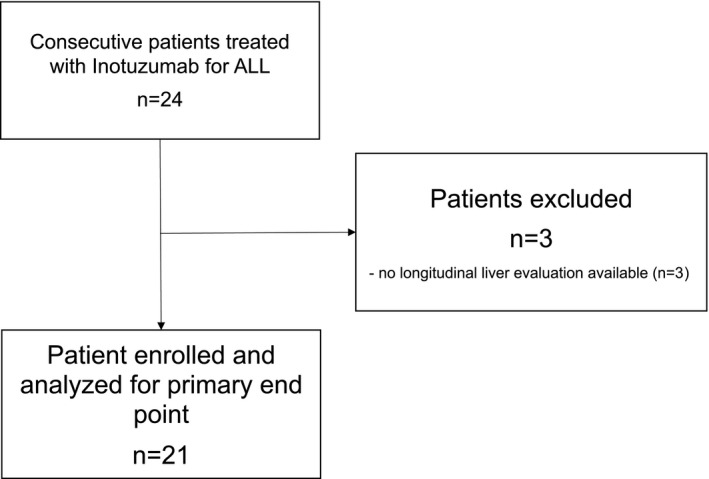
Flow chart of the enrolled patients

At data cut‐off, 21 June 2020, 24 patients received IO at our institution. Twenty‐one patients received baseline assessment and at least a post‐IO assessment included in this study (Figure 1). In our cohort, the median age was 50 years (IQR 33–65); when receiving IO, 16/21 (76%) patients had a relapse of ALL, and 5/21 (24%) patients were refractory after a precedent line of therapy. Patients received a median of 2 (IQR 1.5–3.0) therapy lines before IO; 7/21 (33%) patients underwent HSCT before IO (1 patient had 1st and 2nd HSCT before IO); baseline laboratory results did not show significant leucocytosis, anemia, or thrombocytopenia; furthermore, laboratory tests of liver function and hepatic necrosis were within the range of normality in most of the patients and comparable with R/R ALL patients in different studies.^6^, ^38^ Baseline echography and liver stiffness measurement were comparable with the cohort of patients tested before HSCT and after chemotherapy in our previous experience^39^(Table 1).

**TABLE 1:** Baseline features of study population

Features	ALL patients treated with IO (*N* = 21)
Male/female, *n* (%)	12 (57%)/9 (43%)
Positive BCR rearrangement, *n* (%)	10 (48%)
Median age (25th–75th), years	50 (33–65)
Median white blood cell count (25th–75th), × 10^6^/ml	3.7 (2.2–7.1)
Median hemoglobin (25th–75th), g/L	101.5 (92.2–116.2)
Median platelets (25th–75th), 10^6^/ml	71.5 (50.0–175.2)
Median serum albumin (25th–75th), g/L	38.8 (35.5–42.3)
Gamma(γ)‐glutamyltransferase (25th–75th), U/L	50.0 (20.0–135.0)
Alkaline phosphatase (25th–75th), U/L	73.0 (56.0–104.5)
Aspartate aminotransferase (25th–75th), U/L	22.0 (16.5–36.5)
Alanine aminotransferase (25th–75th), U/L	34.0 (18.0–64.0)
Total bilirubin (25th–75th), mg/dl	0.5 (0.4–0.7)
BMI (25th–75th), kg/m^2^	27.2 (22.8–29.3)
Previous lines of therapy, *n* (25th–75th)	2.0 (1.5–3.0)
Patients who received previous allogeneic stem cell transplant(s), *n* (%)	7 (33%)^a^

Abbreviations: ALL, acute lymphoblastic leukemia; BCR, B‐cell receptor; BMI, body mass index; cm, centimeter; IO, inotuzumab ozogamicin; IQR, interquartile range; N, numbers; SD, standard deviation.

^a^ One patient received two previous allogeneic stem cell transplants.

With a median follow‐up of 17.5 months (IQR 6.0–26.6), patients received a median of six IO administrations, that is, two cycles (IQR 6–12 administrations). Seven out of 21 patients (33%) underwent HSCT after IO therapy (1 patient had a 2nd HSCT after IO). During our study, 11 (52.4%) patients died, primarily due to ALL relapse (10 pts.); 1 patient died in complete remission for complications after HSCT. A clinical diagnosis of VOD/SOS was formulated in two patients (28.6%) after HSCT, and the VOD/SOS was graded as mild and severe, respectively. Clinically relevant complications were reported in a few patients and were inconstantly related to IO therapy (Table S1).

### 3.2 Biochemical changes during and after IO cycles

**FIGURE 2:**
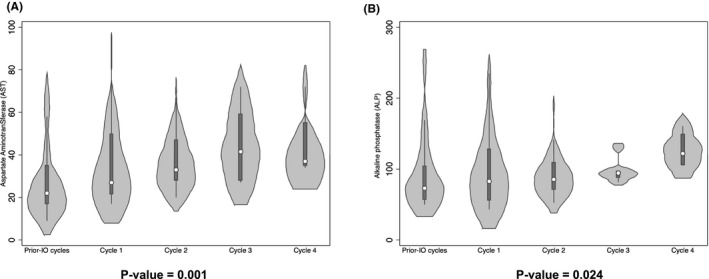
Violin plots and of significant changes in the biochemical parameters of the liver (A‐aspartate aminotransferase [AST]; B‐alkaline phosphatase [ALP])

Among liver biochemical parameters assessed before IO administration and at the end of each IO cycle, aspartate aminotransferase (AST) and alkaline phosphatase (ALP) significantly increased their values cycle after cycle (Figure 2). However, these biochemicals occasionally reached the upper limit of normality (AST > 40 U/L and ALP > 100 U/L) only after 3–4 courses of IO administration. All other liver biochemical parameters analyzed, such as albumin, total bilirubin, γ‐glutamyl transferase (GGT), and alanine aminotransferase (ALT), showed inconsistent variations without achieving significant changes between IO cycles (Supporting Information Figure S1).

### 3.3 Kinetics of measurement of liver stiffness in the different phases of IO therapy

**FIGURE 3:**
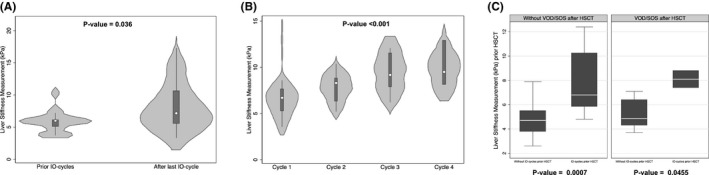
Violin plot and boxplot of liver stiffness measurement kinetics (A) before and after IO therapy; (B) during the IO cycles; and (C) before HSCT and by the occurrence of VOD/SOS after HSCT

Figure 3 shows the kinetics of LSM during the different phases of treatment with IO represented by violin plot and boxplot. Comparing the LSM values before IO therapy (median 6 kPa [5.1–6.1]) with those evaluated at the end of the patient's last IO cycle (7.2 kPa [5.6–10.6]), a statistically significant increase (*p*‐value = 0.036) has been observed (Figure 3A). Patients showed a significant (*p*‐value < 0.001) increase in median LSM cycle after cycle (6.7 kPa [5.3–7.7], 8.3 kPa [6.4–8.8], 9.2 kPa [7.9–11.5], and 9.5 kPa [8.2–12.9], respectively) reaching values > 8 kPa at the end of the second cycle of IO administration with a dose‐dependent kinetic pattern (Figure 3B). When we compared patients treated with IO and subsequently undergoing HSCT with those directly undergoing HSCT (Figure 3C), it was observed that the pre‐HSCT LSM values were significantly higher both in the group that has developed SOS/VOD after HSCT (*p*‐value = 0.0455) and in the group that has not developed SOS/VOD after HSCT (*p*‐value = 0.0007). Although its trend is pointed out (Supporting Information Figure S2), there was no significant difference between LSM values before HSCT in patients who developed SOS/VOD and received prior therapy cycles with IO. No significant differences in these groups were found when liver biochemicals such as AST and ALP were analyzed (Supporting Information Figure S3).

### 3.4 Hepato‐biliary and portal hypertension‐related alterations

**FIGURE 4:**
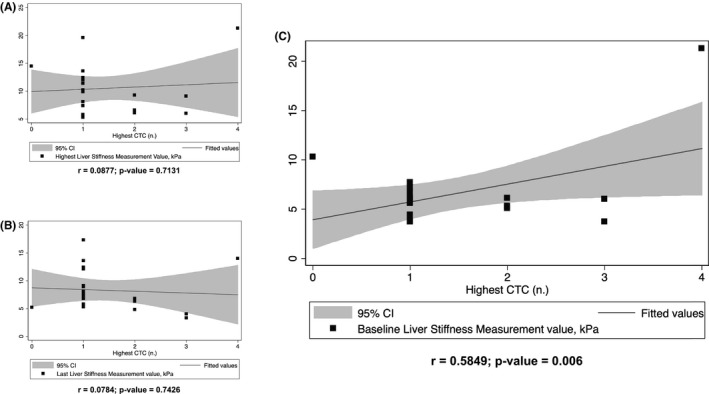
Regression plot of the Spearman rank correlation between highest hepatobiliary complications according to CTCAE grade reached by patients and (A) highest liver stiffness measurement (LSM) value achieved; (B) last LSM value assessed after IO treatment, and (C) baseline (prior IO therapy) LSM value

Table 2 reports the occurrence of liver alterations related to IO therapy during the study period. After evaluating the maximum values reached by patients in each biochemical parameter, following the CTCAE stratification, almost all the patients showed mild hepatobiliary alterations (grade 1). Less than 5% of patients did not experience hepatobiliary alterations. These complications were linked to hepatotoxicity directly related to hepatocellular damage (as demonstrated by the increase in hepatic cytonecrosis parameters such as AST/ALT) and the cholestatic injury caused by the drug (as evidenced by hyperbilirubinemia and increased ALP). Moderate to severe hepatobiliary alterations (grades 2–4) have been observed in nearly one‐quarter of patients treated with IO. A significant positive correlation (Figure 4C) between the highest grade reached by patients in hepatobiliary alterations and baseline LSM values (prior IO therapy) was found (*r* = 0.5849, *p* = 0.006); no significant correlation was found instead with the highest LSM value reached (Figure 4A) and the last LSM value evaluated after the IO treatment (Figure 4B).

**TABLE 2:** Summary of liver complications after IO cycles

	ALL patients treated with IO (*N* = 21)
**(A) Hepato‐biliary complications by CTC**	
No complications	1/21 (4.8%)
CTC≥1	20/21 (95.2%)
Alanine aminotransferase	11 (52.4%)
Aspartate aminotransferase	14 (66.7%)
γ‐glutamyltransferase	12 (57.1%)
Hyperbilirubinemia	2 (9.5%)
CTC≥2	6/21 (28.6%)
Alanine aminotransferase	1 (4.8%)
Aspartate aminotransferase	0
γ‐glutamyltransferase	5 (23.8%)
Hyperbilirubinemia	0
**(B) Portal hypertension‐related complications**	
Hepatomegaly	6 (28.6%)
Splenomegaly	7 (33.3%)
Gallbladder wall thickening >6 mm	1 (4.8%)
Portal vein diameter >12 mm	2 (9.5%)
Portal flows mean velocity <10 cm/s or hepatofugal flow	2 (9.5%)
Mono/bi‐phasic flow or no flow recorded in hepatic veins	4 (19%)
Visualization of paraumbilical vein	1 (4.8%)
Ascites	3 (14.3%)
CSPH according to LSM ≥21 kPa or SSM ≥52.8 kPa	8 (38.1%)
SOS/VOD after HSCT (*n* = 7 pts)	2 (28.6%)
PH‐related signs (*n*)	
Absence (0)	7 (33.3%)
Presence (≥1)	14 (77.7%)
Presence (1–2)	9 (64.3%)
Presence (≥3)	5 (35.7%)

Abbreviations: ALL, acute lymphoblastic leukemia; CSPH, clinically significant portal hypertension; CTC, common terminology criteria; IO, inotuzumab ozogamicin; kPa, kilopascal; LSM, liver stiffness measurement; mm, millimeter; N, numbers; PH, portal hypertension; SSM, spleen stiffness measurement.

**FIGURE 5:**
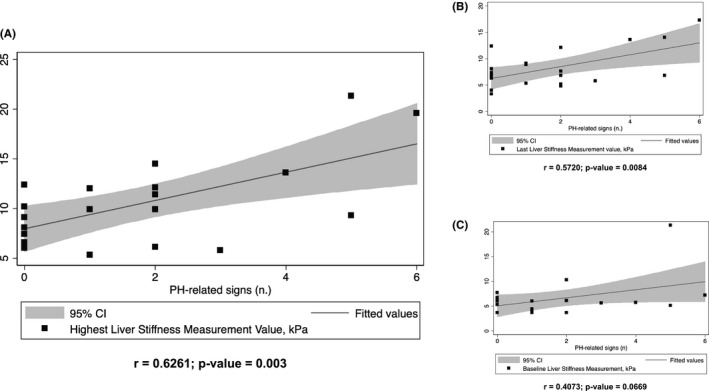
Regression plot of the Spearman rank correlation between number of PH‐related signs developed by patients and (A) highest liver stiffness measurement (LSM) value achieved; (B) last LSM value assessed after IO treatment, and (C) baseline (prior IO therapy) LSM value

Through a noninvasive evaluation based on ultrasound techniques (US color Doppler and transient elastography), we observed that at the end of the IO cycles (Table 2), about one‐third of the patients presented with hepato‐splenomegaly; nearly a quarter of the patients had the US color Doppler features related to portal hypertension, and about 15% of patients developed ascites (of any grade). One patient developed esophageal varices (F2) treated with variceal ligation. According to the noninvasive definition of CSPH based on LSM (≥21 kPa) and SSM (≥52.8 kPa), almost 40% of patients had CSPH at the end of treatment with IO. Counting individually and adding each PH‐related sign collectively, 77% of patients experienced at least one PH‐related alteration. A significant positive correlation between the total number of PH‐related signs developed by patients and the highest LSM value achieved (Figure 5A), the last LSM value evaluated after IO treatment (Figure 5B), and baseline LSM values (prior IO therapy—Figure 5C) were found with an *r* = 0.6261, 0.5720, and 0.4073, respectively. Baseline AST values were not correlated with the higher hepatobiliary alterations based on the grade achieved by the patients (Supporting Information Figure S4A) or the number of PH‐related signs (Supporting Information Figure S4B).

Considering deaths occurred within 60 days from the last IO dose (4), there was no significant difference in the highest LSM value achieved between dead and still alive patients during IO therapy follow‐up (Supporting Information Figure S5).

## 4 DISCUSSION

Since drug approval studies,^6^ IO treatment of relapsed or refractory B‐cell precursors has shown a noteworthy relationship between treated patients and liver complications that could range from simple biochemical alteration associated with hepatocellular damage to actual clinical syndromes related to portal hypertension.^5^, ^33^, ^40^ This study evaluated patients treated with IO followed at our center by a dense hepatic monitoring program through clinical, laboratory, and noninvasive hepatic methods (LSM and US).

We observed that at the end of the IO courses, most of all, the patients had some hepato‐biliary alteration according to CTCAE classification (Table 2), and the baseline LSM was correlated with the development of CTCAE grade. In line with the results of phase 3 INO‐VATE study,^40^ most of the patients had experienced an alteration of some liver biochemical laboratory tests after the IO cycle and only a quarter of these developed a significant hepatobiliary alteration (grade ≥ 2). Although these alterations, among the different liver biochemical tests studied, only AST and ALP showed a significant cycle‐by‐cycle increase with drug‐dependent accumulation kinetics‐like (Figure 2).^41^ Many drugs can cause transient changes in liver biochemical tests. Most adverse reactions are idiosyncratic due to individual patient differences in susceptibility to drug‐induced liver injury (DILI) or inability to recover from injury.^42^, ^43^ Disposal of a calicheamicin metabolite by hepatocytes and subsequent biliary excretion is a possible mechanism of drug‐induced liver injury in this context.^5^ The liver alterations expressed by CTCAE grade were significantly correlated just with prior‐IO LSM assessment (Figure 4C) and not with the highest LSM value reached or the last LSM value evaluated after the IO treatment (Figure 4A,B). This result confirms what we recently observed in a cohort of 78 patients undergoing HSCT,^18^ in whom LSM was able to differentiate the CTCAE grade, allowing us to speculate that hepatic status is more important than the acute IO‐related insult in developing clinically relevant complications. Thus, the baseline LSM can be considered a surrogate for an average of risk factors and previous liver damage.

To the best of our knowledge, with this study, we first monitored PH‐related signs developed by patients treated with IO by a noninvasive evaluation based on ultrasound techniques (US color Doppler and measurement of liver and spleen stiffness by transient elastography). Comparing the liver imaging before and after IO treatment, we observed (Table 2) that around 80% of those developed signs related to PH (i.e., hepatosplenomegaly, hepatic veins flow changes, ascites, and paraumbilical vein visualization). Since the 1990s, abdominal ultrasound's role in associating specific signs of portal hypertension in patients suffering from chronic liver disease^32^ and in the hematological application^15^ has been widely established. These ultrasound findings were also correlated with the diagnosis and prognosis of SOS/VOD post‐HSCT.^26^, ^44^, ^45^, ^46^ Regarding the most fearful PH‐related complication, no patient developed SOS/VOD during treatment with IO (or in the follow‐up without HSCT intervention); however, consistent with the previous reports,^6^, ^33^ the frequency of SOS/VOD after IO and subsequent HSCT was around 30% (Table 2).

The measurement of liver stiffness through TE is the gold standard surrogate to date for assessing the degree of liver fibrosis in the context of chronic liver disease; TE has been extensively studied as a surrogate marker of PH and its complications.^24^ Studies and meta‐analyses have confirmed the close relationship between LSM and HVPG, the gold standard for PH assessment, in patients with ACLD. Liver stiffness has been demonstrated to result from different pathophysiological mechanisms such as inflammation, cholestasis, or increased congestion within the parenchyma.^47^ For these reasons, LSM increased after each dose of IO and would explain the mechanisms underlying liver toxicity and severity of post‐IO portal hypertension. Moreover, the role of these NITs in predicting SOS/VOD and post‐HSCT hepatic complications in different patient cohorts has recently been successfully studied.^16^ Here, using a cohort of patients directly undergoing HSCT as a comparison, it was found that, although pre‐HSCT baseline values were significantly higher in the IO group, these values did not significantly stratify those who subsequently developed SOS/VOD after HSCT (Figure 3C).

The main finding of the study is that mirroring IO‐related liver damage and congestions, the LSM values significantly increase cycle by cycles with median LSM value remarkably higher at the end of the last IO cycle (Figure 3A,B). Furthermore, unlike biochemical tests (Supporting Information Figure S3B), the LSM increases were directly correlated with the number of PH‐related signs at the end of IO courses (Figure 5). The potential mechanisms that could explain the increase in LSM following hepatic injury IO would be differently related (i) to the secondary damage to the targeting of liver sinusoidal cells expressing the antigen expressed on malignant leukemia cells (CD22) and (ii) to the possible presence of cells malignancies within the hepatic vessels with consequent release of toxins and finally hepatic exposure to the cytotoxic metabolite (N‐Ac‐γ‐calicheamicin dimethyl hydrazine).^5^ All these mechanisms could contribute, secondarily to prolonged exposure to IO, to direct and acute hepatocyte damage with consequent deposition of extracellular matrix and formation of hepatic fibrosis, resulting (due to the sinusoidal involvements) in a gradual development of portal hypertension and consequently PH signs and complications. These results are significant because, for the first time, we have identified that noninvasive hepatic monitoring through ultrasound and elastographic evaluations can take over time the onset of possible hepatic complications in patients undergoing a necessary therapy for patients with relapsed or refractory ALL. Given the significant prevalence of hepatic complications reported here and in recent evidence,^33^, ^40^, ^48^ the routine use of LSM and ultrasound before and at the end of each cycle of IO could lead the hematologist in managing the tailored administration of IO (doses, frequency, and the number of infusions per cycle) and in post‐IO follow‐up planning. Indeed, in patients treated with IO, there are basically two follow‐up scenarios; on the one hand, the patient undergoing post‐IO HSCT and on the other hand, the patient who is not eligible for HSCT and who must be followed until death.^49^

In the first case, it should be noted that the number of patients who developed SOS/VOD among those who underwent HSCT (2/7) was presumably too small for the formal statistical test and definitive conclusion (Supporting Information Figure S2). Although statistical significance was not reached in our study, the median LSM values indicative of post‐IO SOS/VOD are likely to be higher than those did not previously treated with IO.

However, given the higher incidence of post‐IO HSCT SOS/VOD, patients would benefit from a pre‐HSCT assessment that considers the patient's different risk factors and adjusts conditioning regimens accordingly.^50^, ^51^ Furthermore, similar to what has been recently described in pediatric^19^, ^52^ and adult populations^18^, ^23^ of patients undergoing HSCT, the bedside LSM monitoring would seem to allow a pre‐clinical diagnosis of SOS/VOD and differential diagnostics with other liver complications.^18^, ^23^

On the other hand, our results showed that patients followed up without HSCT referrals die in the following months, mainly for ALL‐related events. However, given the presence of long‐term survivors (Supporting Information Table S1) and the significant number of PH‐related signs developed (Table 2), these patients may benefit from conventional clinical and imaging follow‐up of a patient with compensated ACLD (also known as cirrhosis of the liver).^17^ Consequently, screening and prevention of PH‐related complications such as refractory ascites^53^ and esophageal varices and variceal bleeding^54^ are crucial. Indeed, these complications could significantly impact the quality of life and overall survival of these patients. The development of such complications related to a chronic PH status in a patient treated with IO has similarly been described as a consequence of long‐term oxaliplatin‐based chemotherapy in colorectal cancer patients.^12^, ^55^, ^56^, ^57^, ^58^ Hepatic involvement secondary to oxaliplatin‐based chemotherapy likewise IO therapy could fall into a recently described classification called portal sinusoidal vascular disease (PSVD).^58^ PSVD is characterized by typical manifestations of PH (appearance of esophageal varices, ascites, and portosystemic collaterals) and could probably explain the vascular and inflammatory hepatic changes identified in our study.

To the best of our knowledge, this is the first study that has the strength to perform a comprehensive laboratory and liver imaging (ultrasound and LSM) evaluation of hepatobiliary complications in patients undergoing IO treatment using an intensive cycle‐by‐cycle program.

However, the present study has some limitations. A relatively small cohort of patients was included, albeit to the best of our knowledge, this is the first single‐center study on this topic. The absence of a parallel control group did not allow us to exclude with certainty that some of the observed variations are secondary to late effects of previous therapy or other confounding factors. Moreover, nevertheless few patients subsequently undergoing HSCT were included, the prevalence of SOS/VOD was in line with what has been reported in the literature.^33^ Noninvasive liver assessments were arbitrarily scheduled before and after each cycle of IO. Evidence for a standard assessment program is not yet available, and our program has been arbitrarily applied due to the experimental role of these techniques in this context. Another possible limitation of our study is the lack of histological evaluation of the liver; however, liver biopsy is difficult to perform in this setting, mainly in patients with impaired coagulation and low platelet counts. Finally, our patient population was evaluated for a baseline risk factor for liver complications (e.g., previous therapies, previous HSCT, iron load, etc.); however, the number of our patient sets did not allow stratification for the analyses.

Based on our experience, future research should focus on confirming the predictive role of liver imaging on liver complications and the impact of these strategies on the overall survival of patients treated with IO in cohorts (hopefully multicenter), more extensive with a more extended follow‐up period.

In conclusion, this monocentric, prospective, longitudinal study on hepatic evaluation of patients receiving label IO suggests that a noninvasive assessment by color Doppler ultrasound and liver stiffness measurements may be promising approaches for diagnosis and monitoring portal hypertension‐related complications after IO treatment.

## CONFLICT OF INTEREST

AC has received honoraria for participation in advisory boards from Jazz Pharma; GMart acts as a consultant for ARIAD/INCYTE, Jensen, Pfizer, Celgene/BMS, Amgen, J&J, and Roche; The remaining authors declare no competing financial interests.

## AUTHOR CONTRIBUTIONS

FR, GMarc, AC, and CP designed the research and analyzed the data, and all authors contributed to data collection and wrote the manuscript. All authors had access to the data and were involved in this paper's analysis, interpretation, review, and preparation.

## Supporting information

Fig S1

Fig S2

Fig S3

Fig S4

Fig S5

Table S1

## Data Availability

For original data, please contact the antonio.colecchia@unimore.it
