# Supplementary figures and images for: Assessment of liver stiffness measurement and ultrasound findings change during inotuzumab ozogamicin cycles for relapsed or refractory acute lymphoblastic leukemia
Source: Cancer Med. 2021 Dec 30;11(3):618–29. doi: 10.1002/cam4.4390 (PMC8817094; doi:10.1002/cam4.4390)

**A**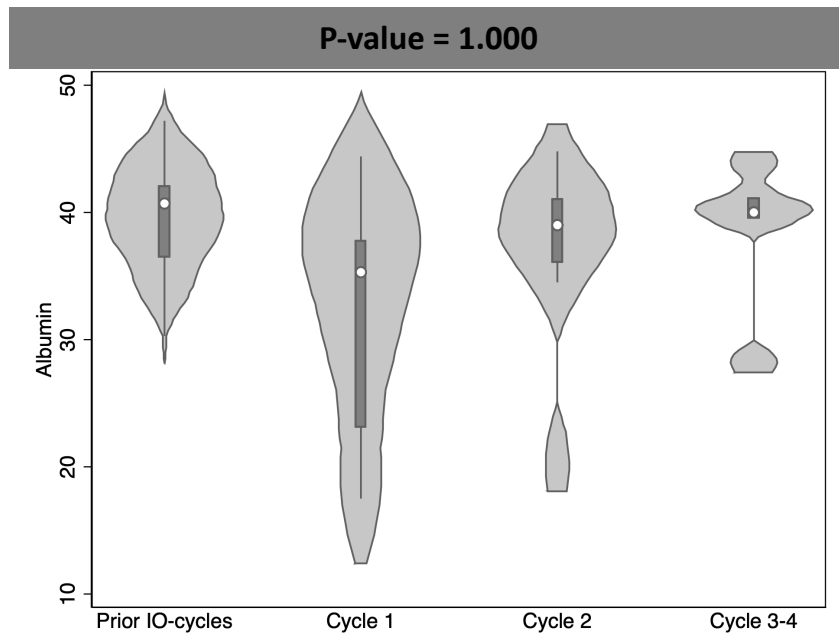**B**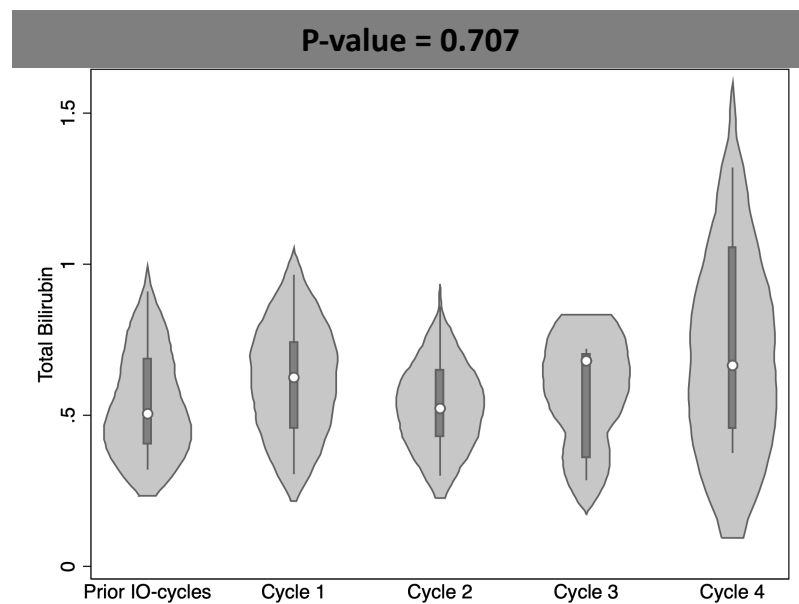**C**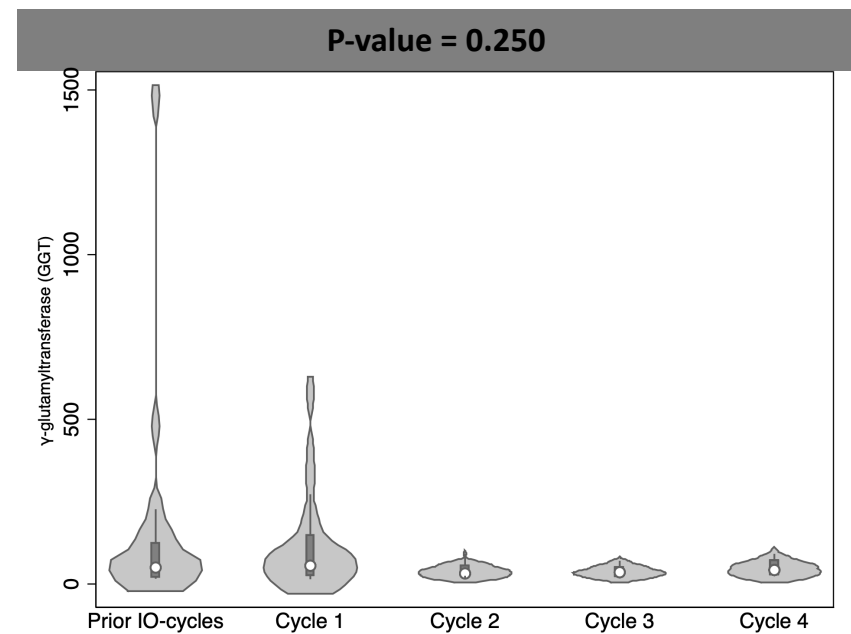**D**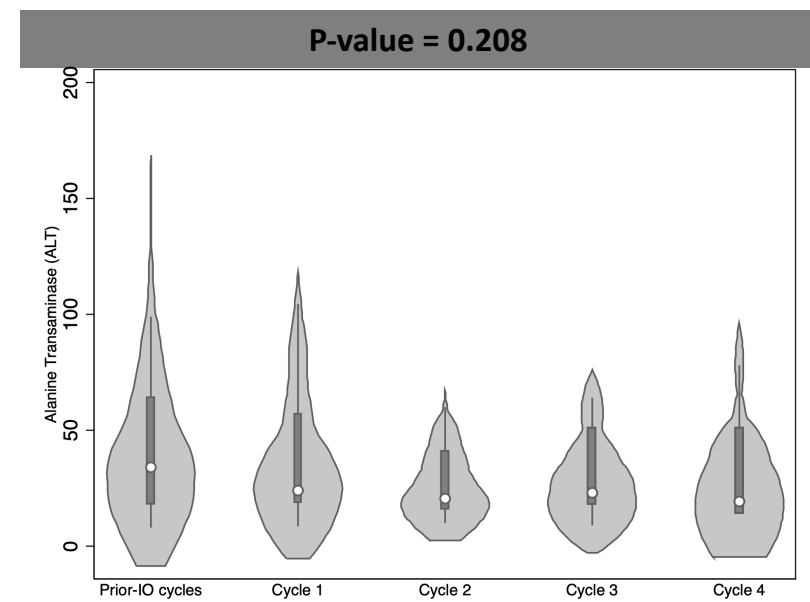

Supplement: Supplementary file 1 — Fig S1 [file CAM4-11-618-s003.pdf]

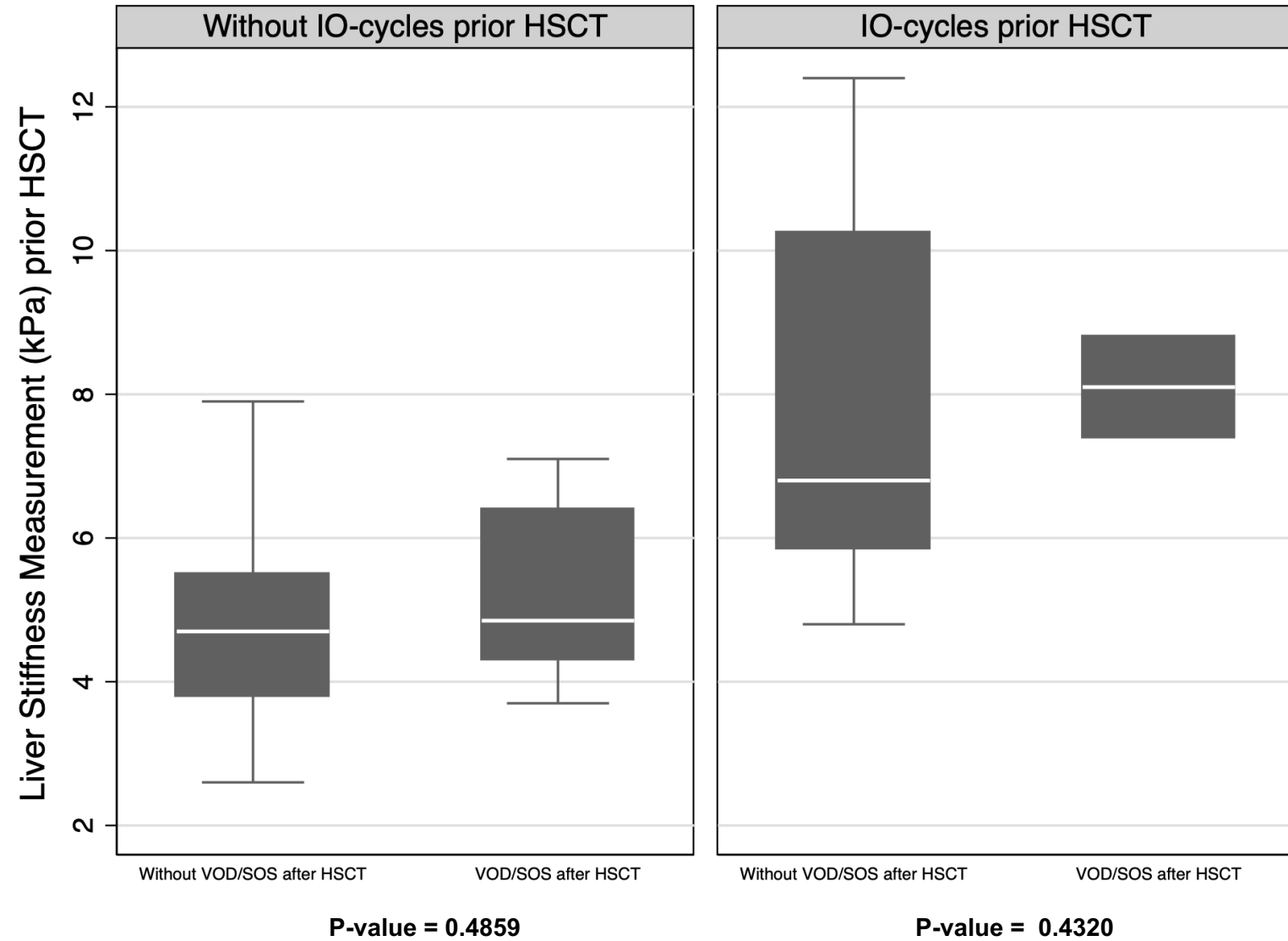

Supplement: Supplementary file 2 — Fig S2 [file CAM4-11-618-s006.pdf]

**A**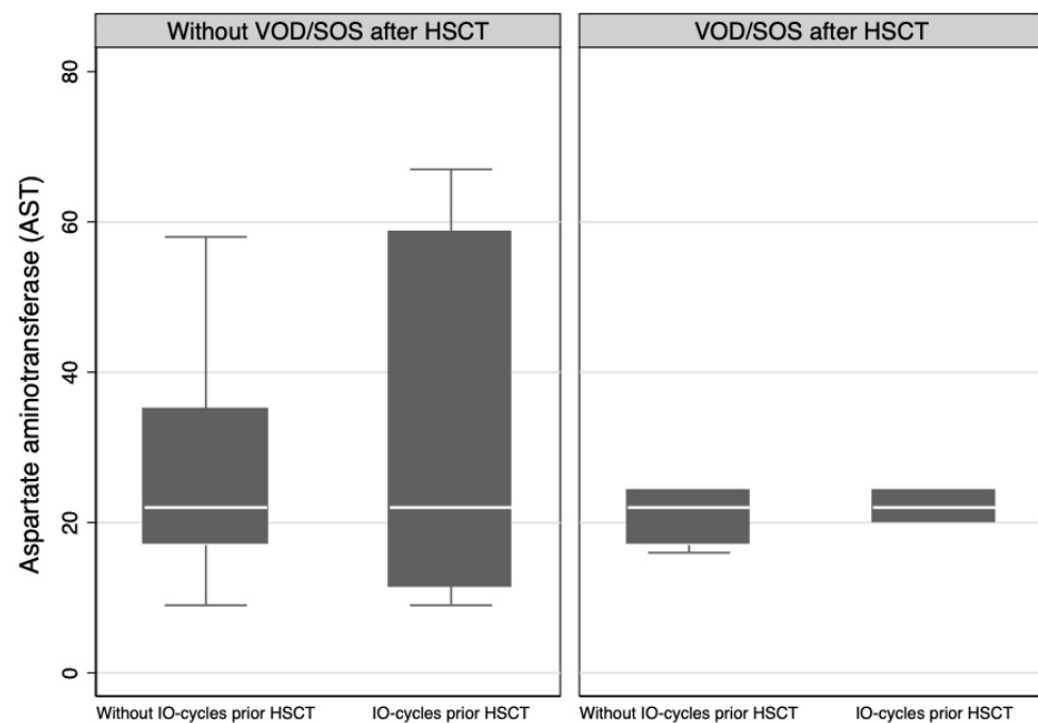**P-value = 0.9464****P-value = 0.8635****B**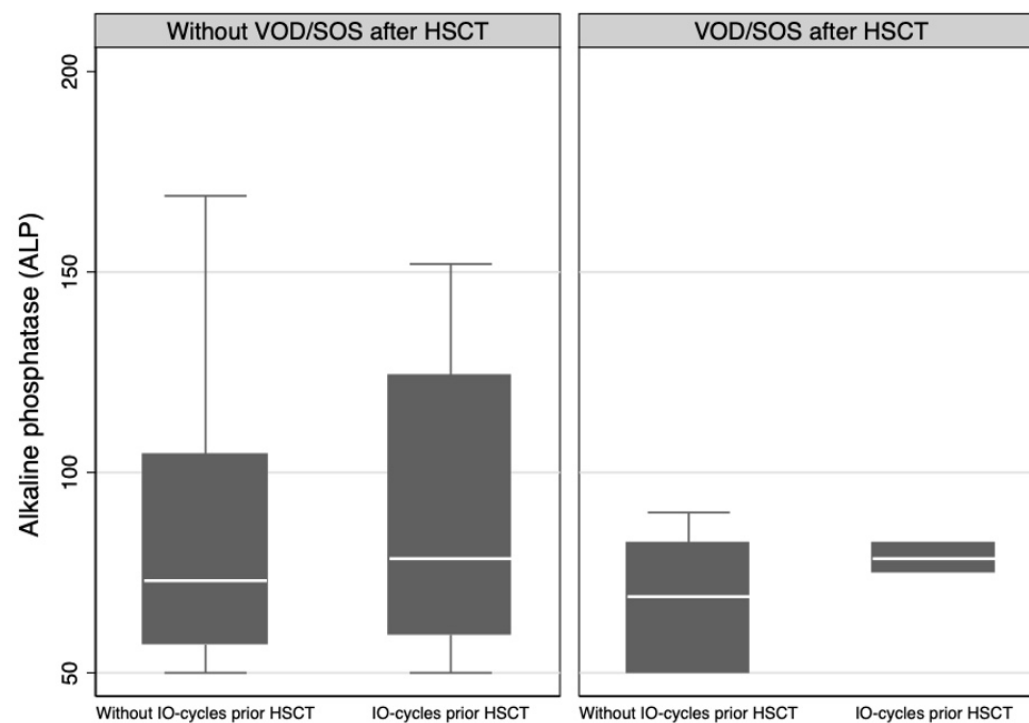**P-value = 0.7997****P-value = 0.3990**

Supplement: Supplementary file 3 — Fig S3 [file CAM4-11-618-s002.pdf]

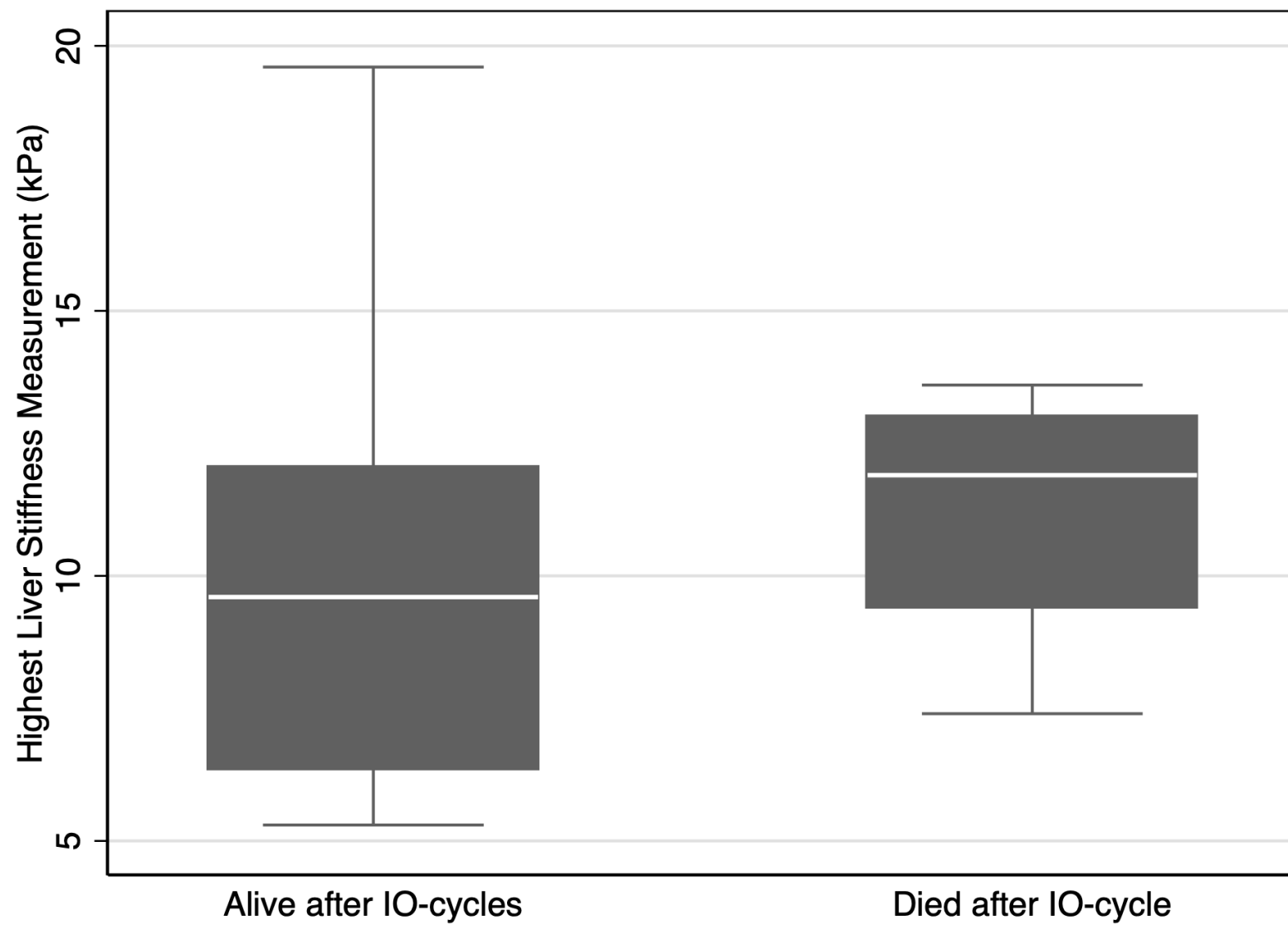

**P-value = 0.3445**

Supplement: Supplementary file 4 — Fig S4 [file CAM4-11-618-s005.pdf]

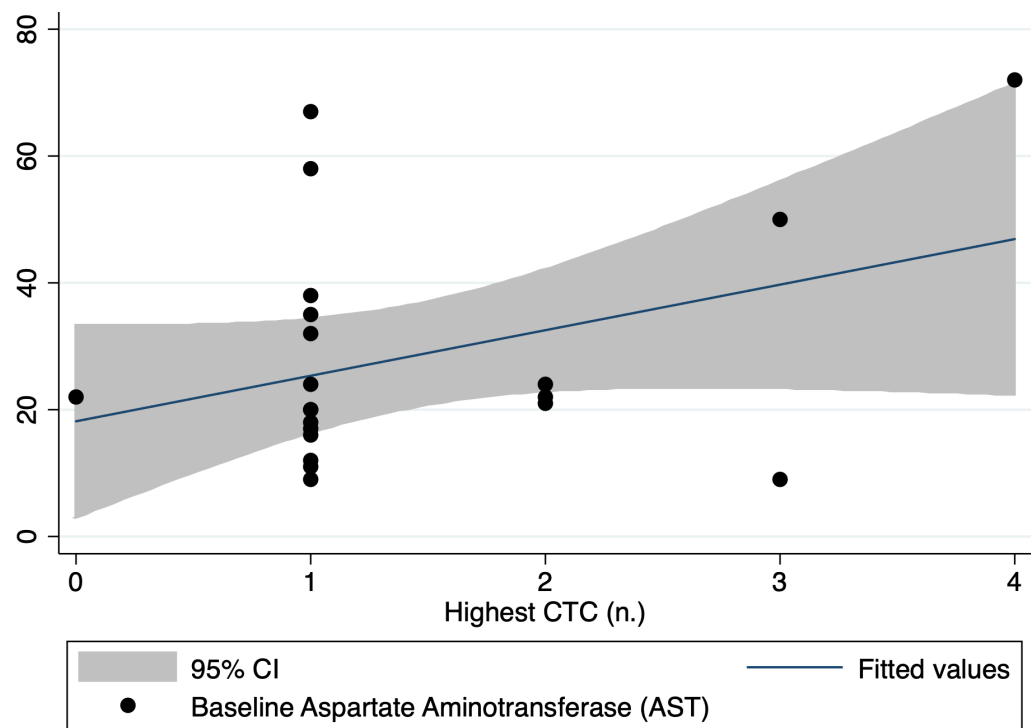

$r = 0.3577$ ; p-value = 0.1114

A

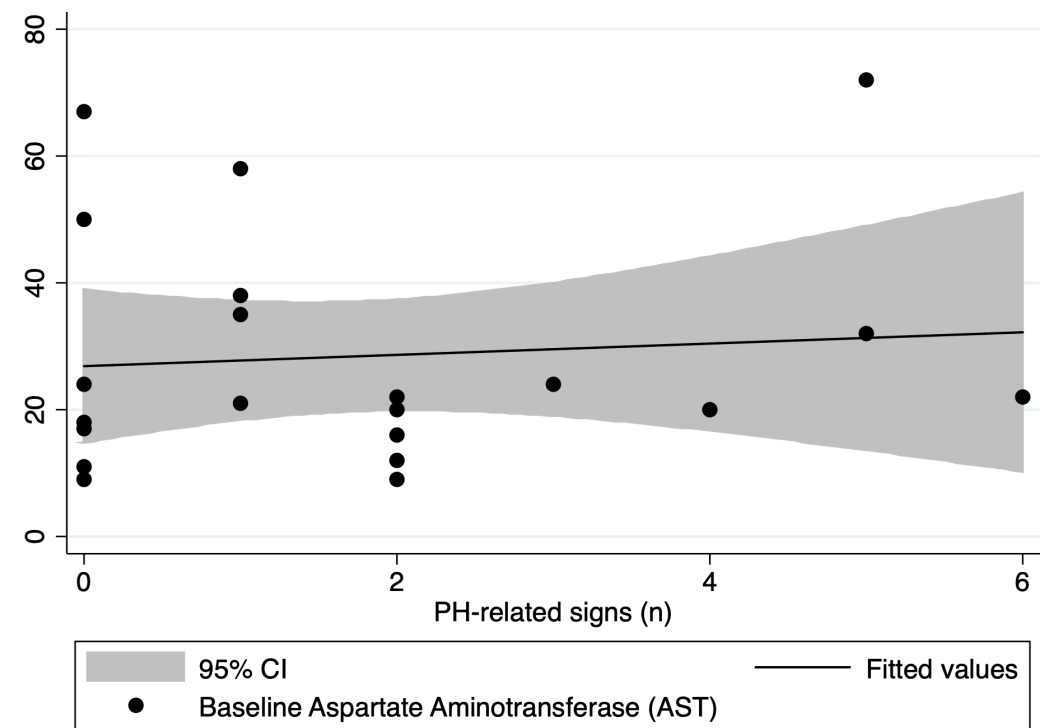

$r = 0.0894$ ; p-value = 0.6998

B

Supplement: Supplementary file 5 — Fig S5 [file CAM4-11-618-s004.pdf]
