# Supplementary material for: Assessment of liver stiffness measurement and ultrasound findings change during inotuzumab ozogamicin cycles for relapsed or refractory acute lymphoblastic leukemia
Source: Cancer Med. 2021 Dec 30;11(3):618–29. doi: 10.1002/cam4.4390 (PMC8817094; doi:10.1002/cam4.4390)
Supplement: Supplementary file 6 — Table S1 [file CAM4-11-618-s001.docx]

**Supplemental Table 01: Clinical outcome and liver complication at patient level.**

| Patient | Last follow-up; status [time after the end IO therapy] | Clinical hepatologic manifestation; [time after the end IO therapy] | Max CTC reached in among hepatobiliary complications | Occurrence of ultrasound portal-hypertension findings |
| --- | --- | --- | --- | --- |
| 1 | Dead [2m]. *Died in CR after HSCT for alveolar hemorrhage and MOFS. ** | VOD, grade severe, after HSCT [2m] | CTC 1 | Yes |
| 2 | Alive in CR [44m]. | cGVHD of the liver (18 m post INO, 26 m post HSCT)), grade moderate (I) and esophageal varix (F1-F2) treated with variceal ligation | CTC 2 | Yes |
| 3 | Dead [9m]. *Disease progression. ** | Leukemia involvement of the liver with ascites (diagnosed on biopsy), not related to IO [8m] | CTC 1 | Yes |
| 4 | Dead [1m]. *Disease progression* | / | CTC 3 | Yes |
| 5 | Dead [17m]. *Disease progression.* | / | CTC 1 | Yes |
| 6 | Dead [19m]. *Disease progression* | / | CTC 3 | Yes |
| 7 | Alive in CR [37m]. *** | VOD, grade mild, after HSCT [3m]. | CTC 1 | Yes |
| 8 | Dead [18m]. *Disease progression.* | / | CTC 1 | No |
| 9 | Alive in CR [36m]. | / | CTC 1 | No |
| 10 | Alive after disease relapse [35m]. | / | CTC 1 | Yes |
| 11 | Dead [18m]. *Disease progression.* | / | CTC 2 | Yes |
| 12 | Alive after disease relapse [29m]. * | cGVHD liver grade I/II after HSCT | CTC 1 | Yes |
| 13 | Alive after disease relapse [27m]. * | / | CTC 2 | Yes |
| 14 | Dead [4m]. *Disease progression.* | / | CTC 1 | No |
| 15 | Dead [6m]. *Disease progression.* | Hyperammonemia, grade mild [4m]. | CTC 1 | Yes |
| 16 | Alive in CR [23m] | / | CTC 1 | No |
| 17 | Dead [6m]. *Disease progression.* | / | CTC 1 | Yes |
| 18 | Dead [15m]. I*n CR after HSCT due to covid-19 infection. ** | / | CTC 1 | No |
| 19 | Alive in CR [25m]. | / | CTC 4 | Yes |
| 20 | Alive during Blinatumamab therapy, no response to IO | Leukemia involvement of the spleen and the liver [0m] | CTC 1 | No |
| 21 | Alive during IO therapy [2m]. | / | CTC 0 | No |

* received HSCT after IO
